# A polymorphism at codon 31 of gene p21 is not associated with primary open angle glaucoma in Caucasians

**DOI:** 10.1186/1471-2415-5-5

**Published:** 2005-04-04

**Authors:** Thomas Ressiniotis, Philip G Griffiths, Sharon M Keers, Patrick F Chinnery, Michael Birch

**Affiliations:** 1 Department of Ophthalmology, Royal Victoria Infirmary, Newcastle upon Tyne, UK; 2 Department of Neurology, The Medical School, The University of Newcastle upon Tyne, UK

## Abstract

**Background:**

Primary open angle glaucoma (POAG) is considered to be a neurodegenerative optic neuropathy, in which cell death occurs by apoptosis. *p21*, is an important protective component of the apoptotic pathway, regulating cellular arrest in the presence of DNA damage. An unstable or altered p21 protein could modify the cellular response to genomic injury and abolish the effect of *p21*. A previous study on a Chinese cohort suggested that the *p21 *codon 31 polymorphism may alter the state of apoptosis in glaucomatous optic neuropathy, failing to protect the ganglion cells. The aim of this study was to test the hypothesis that a *p21 *codon 31 polymorphism is associated with POAG on a Caucasian cohort.

**Methods:**

140 POAG patients and a control group of 73 healthy individuals were included in the study. All the subjects were of Caucasian origin. Genomic DNA was amplified by polymerase chain reaction, followed by enzymatic restriction fragment length polymorphism technique (PCR-RFLP). Patients and controls were genotyped for a single nucleotide polymorphism (C/A transversion) in the third base of codon 31 of p21, which leads to a serine (Ser)/arginine (Arg) substitution.

**Results:**

The distribution of the genotypes in the POAG patients showed 128 (91.4%) Ser homozygotes, 10 (7.1%) Ser/Arg heterozygotes and 2 (1.5%) Arg homozygotes. In the control cohort, there were 61 (83.6%) Ser homozygotes and 12 (16.4%) Ser/Arg heterozygotes. No Arg homozygotes were present amongst the control group. Both the allelic and genotypic frequencies of the Ser or Arg residues at codon 31 were not significantly different between POAG patients and controls (Fisher's exact test, P = 0.20 for alleles and P = 0.0561 for genotypes).

**Conclusion:**

This study suggests that the p21 codon 31 polymorphism does not contribute to the risk of POAG in the Caucasian population.

## Background

Primary open angle glaucoma (POAG [MIM 137760]) is a multifactorial neurodegenerative disease, causing blindness to approximately 70 million individuals worldwide [1,2]. The aetiology of POAG is poorly understood, with both genetic and environmental factors contributing to the pathophysiology [3,4] Irrespectively of the causative factors leading to visual loss, most of which are still not understood, the final common pathway in POAG is retinal ganglion cell death, mediated by apoptosis, a genetically regulated process [5].

The apoptotic pathway consists of multiple interacting pathways, some of which are still unclear. It appears that, following DNA damage, cells can either proceed to apoptosis or enter a transient arrest cycle, allowing time for DNA repair. *p21 *gene, also known as *WAF1 *or *CIP1*, is a key component of this pathway. It can be up-regulated either by activated wild type p53, which acts as a transcription factor [6], or independently, by various factors such as TGF*β*, vitamin D, TPA and nerve growth factor. *p21 *expression results in inhibition of the cyclin-dependent kinases (Cdks), that are essential for cell division. Consequently, cell cycle is arrested at the G1 phase, until genomic repair is established.

An unstable or altered p21 protein, therefore, could significantly affect the activity of Cdks, modifying the cellular response to genomic injury and abolishing the protective effect of p21. Such an outcome can derive from a single nucleotide polymorphism in the third base of codon 31 of the *p21 *gene, following a C to A transverse change, which results in a Serine/Arginine amino acid substitution. This polymorphism probably encodes a probable DNA-binding zinc-finger domain, causing functional changes to the p21 protein.

There is evidence from a recent case-control study on a Chinese cohort that the Arg allele of the p21 codon 31 polymorphism is more common amongst POAG patients [7]. In this study, we tested the hypothesis of a possible association between the p21 codon 31 polymorphism and POAG on a Caucasian population.

## Methods

### Case selection

Having obtained ethical approval from our local research ethics committee, blood samples were analysed from an unrelated Caucasian cohort of 140 POAG patients and 73 controls from the north east of England.

The definition for POAG included characteristic cupping of the optic disc, open iridocorneal angle and typical glaucomatous visual field defects. An experienced glaucoma specialist clinically examined both patients and control groups (M.B.). Patients with intraocular pressure (IOP) higher than 30 mmHg at first presentation and secondary types of glaucoma (pseudoexfoliative, pigment dispersion syndrome, trauma or steroid induced) were excluded. The control group consisted of the spouses of our POAG patients, who were free from any coexisting ocular pathology and had normal visual acuity, IOP, visual fields and optic discs.

### Molecular genetic analysis

Purified genomic DNA was amplified by polymerase chain reaction (PCR) for exon 2 of the p21 gene. For each subject, 1 μl of DNA was mixed with 1 unit Taq DNA polymerase (Promega, Madison, WI, USA), 10x Taq polymerase buffer (Promega, Madison, WI, USA), 2 mmol dNTP, 0.25 μM of each oligonucleotide (primer) and H_2_O to total volume of 30 μl. The primers used were: Forward (5'-GTC AGA ACC GGC TGG GGA TG -3') and reverse (5'- CTC CTC CCA ACT CAT CCC GG -3'). Reactions were treated in a thermal cycle machine to incubation at 94°C for 5 min followed by 35 cycles of: 94°C for 30 sec, 57.2°C for 30 sec, 72°C for 30 sec and a final incubation of 72°C for 7 min.

For each sample, the amplified PCR product was digested with the restriction enzyme *Blp *I (New England Biolabs, Beverly, Massachusetts, USA). The *Blp *I digestion mixture contained 10 μl PCR product, 6 units of enzyme, 3 μl buffer (NEB Buffer 4) and H_2_O to total volume of 20 μl. The reactions were allowed to proceed for 12 hours at 37°C.

The resulting fragments were separated by electrophoresis on a 3% agarose gel and visualised by ethidium bromide staining with a digital camera. The Ser allele has a single *Blp *I restriction site (GCTNAGC), resulting in two fragments of 89 bp and 183 bp, where the Arg allele remains undigested, producing a single band of 272 bp.

The molecular genetic analysis was performed in the same laboratory by two investigators who were masked to the phenotype of the samples studied.

Fisher's exact test was used to compare the genotype and allele frequencies in cases and controls.

## Results

Our cohort consisted of 140 POAG patients and 73 controls. The median age was 73 years for the POAG patients (range 51–87, S.D. = 8.01) and 78 years for the controls (range 68–90, S.D. = 4.4). Mean IOP was 20.8 mmHg for the patients (S.D. = 2.6) and 16.2 mmHg for the controls (S.D. = 3.4). Median cup/disc ratio was 0.8 and 0.3 for patients and controls respectively. The frequency distribution and the allelic frequencies of p21 codon 31 polymorphisms in POAG patients and healthy subjects are illustrated in Table 1 and 2 respectively. The allele frequencies follow Hardy-Weinberg proportions.

The distribution of the genotypes in the POAG patients was 128 (91.4%) Ser homozygotes, 10 (7.1%) Ser/Arg heterozygotes and 2 (1.5%) Arg homozygotes. In the control cohort, there were 61 (83.6%) Ser homozygotes and 12 (16.4%) Ser/Arg heterozygotes. No Arg homozygotes were present amongst the control group. We therefore pooled the Arg/Arg groups with the Ser/Arg group for the genotype analysis. Both the allelic and genotypic frequencies of the Ser or Arg residues at codon 31 were not significantly different between POAG patients and controls (Fisher's exact test, P = 0.20 for alleles and P = 0.0561 for genotypes).

## Discussion

Apoptosis and cell-cycle arrest are regulated through various interacting pathways [8]. DNA damage leads to activation of transcription factors, such as the *p53 *gene, which can induce apoptosis by up-regulating the protein Bax and down-regulating the protein Bcl-2. Alternatively, DNA insult can cause activation of the *p21 *gene, a tumour suppressor gene, either directly or through transactivation by wild type *p53. p21 *is a cyclin – dependent kinase inhibitor resulting in cell-cycle arrest by inhibiting the G1 to S and S to G2 phases [9]. Cancer research has revealed that mutations of *p21 *are very rare and that single nucleotide polymorphisms are more likely to have a functional effect [10]. The Ser/Arg polymorphism at codon 31 is located in a highly conserved region of the *p21 *[11] and has been associated with cancer of the lungs, breast [12], bladder [13], and colorectal tumours [14].

A recent study on a Chinese population showed an association between the Arg form of the *p21 *codon 31 polymorphism and POAG, suggesting that this allele may alter the state of apoptosis in glaucomatous optic neuropathy, failing to protect the ganglion cells [7].

In our cohort, we have not detected any statistically significant difference of the Ser and Arg allelic frequencies between the POAG group and the healthy individuals. This difference may reflect sampling bias, as the Chinese cohorts were smaller (58 POAG patients and 59 control subjects), or it could be attributed to ethnic disparity. The Arg allele is considerably more common in Chinese, with reported frequency of 0.50. Our findings correlate well with previous studies on Swedish and French populations, which identified a low Arg allele frequency of 0.05 [15].

However, we cannot exclude the possibility of p21 codon 31 polymorphism being associated with POAG in other ethnic groups or the likelihood of an association between POAG and another p21 polymorphism. This might be a worthwhile future strategy to elucidate the issue.

## Pre-publication history

The pre-publication history for this paper can be accessed here:



## Figures and Tables

**Table 1:** Frequency distribution of p21 codon 31 polymorphisms in POAG patients and healthy subjects. "Ser" and "Arg " represent encoding of Serine and Arginine, respectively, from the polymorphic site on exon 2.

	Ser/Ser	Ser/Arg	Arg/Arg	Total
POAG (n = 140)	128 (91.4%)	10 (7.1%)	2 (1.5%)	140
Controls (n = 73)	61 (83.6%)	12 (16.4%)	0 (0.0%)	73
Total	189 (88.8%)	22 (10.3%)	2 (0.9%)	213

Fisher's exact test: The two-tailed P value equals 0.0561. (Note: The frequencies of the Arg homozygotes were excluded from the analysis, as they were very low in the POAG group and 0 in the control group)

**Table 2:** Allelic frequencies in POAG patients and healthy subjects.

	Ser	Arg	Total
POAG	266 (95%)	14 (5%)	280
Controls	134 (91.8%)	12 (8.2%)	146
Total	400	26	426

Fisher's exact test: The two-tailed P value equals 0.20.
